# Biological Invasion Data Gaps in China: Examples of Distribution, Inventories, and Impact

**DOI:** 10.3390/biology13110872

**Published:** 2024-10-26

**Authors:** Jiayao He, Ke Chen, Peishan Sun, Han Xu, Xubin Pan

**Affiliations:** 1 Institute of Plant Inspection and Quarantine, Chinese Academy of Inspection and Quarantine, Beijing 100176, China; 2 Research Center of GACC for International Inspection and Quarantine Standards and Technical Regulations, Beijing 100013, China

**Keywords:** biosecurity, data demands, data gap analysis, proactive management

## Abstract

The impact of invasive alien species (IAS) on nature and society is increasing globally. It is crucial to utilize information systems for evidence-based management, enabling the assessment and supporting survey and control actions. However, the lack of accessible and comprehensive baseline IAS data often impedes the ability to prioritize and allocate resources efficiently. Despite the increased public awareness of biological invasions in China over the past decades, the critical importance of data requirements has not been fully recognized, leading to gaps in available data. Here, we outline the key data demands for the management of biological invasions and highlight the current lack of high-quality data for invasion management in China, and critically assess data gaps in IAS distribution, inventory, and impact. Additionally, we propose a conceptual framework to illustrate the data requirements throughout the invasion management process, along with indicators to assess data quality within three dimensions: relevance, resolution, and reliability.

## Simple Summary

The growing impact of invasive alien species highlights the need for effective management. However, the lack of baseline data on invasive alien species often hinder the efficient allocation of resources. Data requirements for invasive alien species remain under-recognized, leading to insufficient evaluation of the gaps. Our study identifies critical data gaps and proposes recommendations for improving the collection, curation, and quality control of invasive alien species data in China.

## 1. Introduction

The growing threats to biodiversity, ecosystem services, and society sustainability posed by invasive alien species (IAS) underline the need to integrate information and develop assessment approaches on IAS [1,2,3,4]. Accurate and sufficient information on IAS facilitates the management of biological invasions and the ability to respond rapidly. In addition, reliable data provide a basis for discerning invasion patterns and, thus, affect policy-making and management actions related to reducing IAS threats directly [5,6]. These data can be used to prioritize IAS and allocate management resources more objectively [7]. However, comprehensive standardized baseline data have not been available, and there are multiple data gaps despite a substantial increase in invasion research on IAS. 

China encompasses many endemic species and biological resources that are critically vulnerable to a wide range of IAS [8]. Despite many efforts, biological invasions have been a major cause of the accelerated loss of biodiversity. Previous studies have demonstrated more than 660 invasive alien species, according to a government report (2019 Report on the State of National Ecology and Environment). However, this report lacks detailed information on the specific composition of these invasive organisms. In addition, annual economic losses caused by IAS in China have been estimated at more than $18.9 billion, with invasive alien insects accounting for a large proportion of these losses [9]. However, several studies have shown that the number of documented invasive alien species and the economic losses caused by them seem to be underestimated [10,11,12,13]. As global trade has intensified the movement of many species across natural dispersal barriers, non-native species have become naturalized and even invasive [14]. Importantly, trade volume and human footprint are important indicators of biological invasion, and IAS has become a driver of biodiversity threats, particularly in developing countries [15]. China is now suffering more from these threats than at any other time in the past few decades [16].

It is crucial to understand the specific data required at each stage of the biological invasion process to manage invasive alien species, further contributing to sustainable development goals. IAS data that is available, accessible, and of sufficient quality is essential for supporting evidence-based management. However, existing information on IAS data in China, which is fundamentally important to both scientific and political agendas, is currently fragmented. Our study aimed to make a data gap analysis within IAS distribution, inventories, and impact in China, indicating the specific role of data in invasion research and management and then providing insights into the baseline information needed to underpin policy-making and management settings for IAS.

## 2. IAS Data Demands and Gaps

**Figure 1:**
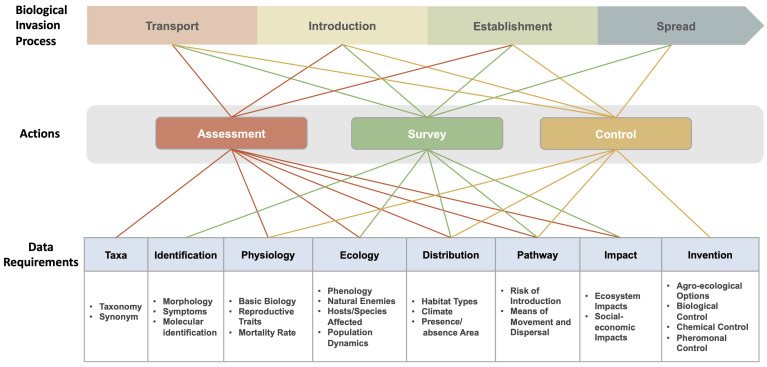
General outline of data required for the stages of invasion and generation of key management actions. Each stage has particular data requirements to ensure effective responses. These data are essential for decision-making, prioritizing resource allocations, and managing biological invasions.

To determine which alien species are “invasive” and to predict the risk of a species being introduced, establishing, spreading, and causing consequences, relevant information regarding its taxonomy, pathways, habitat types, abiotic and biotic factors, and impacts associated with these species, should be required [17]. The availability of the above information can help with evidence-based management and play a vital role in data-driven research in invasion biology [18,19]. In addition, they can provide opportunities for improvements in related analytical approaches and can contribute to an in-depth understanding of invasion mechanisms in the global context [1]. Accordingly, inventories with established numbers and distribution range of IAS allow for an enriched knowledge in invasion patterns [20], while incomplete and unstructured data of biological invasions would get in the way of management efforts [21]. Here, we propose a conceptual framework to illustrate the data requirements throughout the biological invasion process and management actions (Figure 1).

### 2.1. Distribution

Data on spatial and temporal distributions is an essential part of bio-invasion knowledge, which is necessary to estimate existing/potential threats and manage IAS [21]. Interestingly, identifying species introductions from a historical perspective—geographic origins and year of first record—was found even more effective than traits in explaining invasion success along multiple dimensions of invasiveness [22,23]. Historical and current data on species distributions are needed to track changes in biodiversity [24]; however, the reliability of inferences from these records is highly impacted by data quality. Notably, occurrence records are important for examining species’ responses or adaptation to environmental variables. Integrating distribution data and spatial variables can inform assessments, surveys, and control. For example, the year and sites of an IAS first record can infer the primary introduction pathway of this species, which maybe link to accidental escapes from ornamental and horticultural activities, thereby assisting the regulation of IAS gateway areas and improving pathway management [25].

Many government professionals and researchers have regarded “evaluating the quality of distribution data” as the most difficult part of usage for species occurrence information systems [26]. The geographical distribution of the IAS can be compiled at sub-regional administrative divisions. However, various factors such as environmental variables and vegetation cover, that could account for biological invasion are not consistent among these artificially zoned sites. Thus, their applications are difficult owning to such discrepancies. As such, finer distribution data rather than summaries at coarser resolutions are required, and monitoring of invasion trends requires more than a single snapshot of the distribution [24].

High-resolution distribution data are often lacking or only exist at a small spatial or short temporal scale in China, expecially in the remote areas such as marine islands or high elevation mountainous area (Table 1). In several studies, species occurrence has been used to analyze biological traits related to invasion success [27], estimate the spatial extent of invasive patterns [28], and identify climate, land use, and socioeconomic factors that could affect IAS distribution patterns [29,30,31,32]. However, occurrence data have mostly been complied at the county level [33]. Moreover, the occurrences of some high-profile IAS have only been collated at the provincial and county levels [34,35]. In addition, early catalogue that has not been updated for a long time which has often been used in recent research and is likely to undermine the credibility of the results. For example, basic distribution data used in a 2020 study to analyze spatiotemporal patterns were recorded in Ma (2014) [33,36]; however, the introduction and spread of IAS has been rapidly growing in recent years. Despite the effectiveness of technologies and tools such as species distribution models (SDMs) in predicting the potential geographical distribution of IAS, which is useful to inform proactive management strategies, the lack of baseline data at finer distribution resolutions remains one of the key gaps that need to be addressed.

### 2.2. Inventories

IAS inventories from distribution are necessary to provide historical baselines and inform management strategies. Therefore, it is essential to compile inventories of exotic species and develop effective tools for storing IAS data. Ensuring reliability and completeness is a major challenge when facing enormous volumes of data. Additionally, the depth and range of surveillance activities can directly affect the resolution of IAS data. 

Previous studies have shown a positive correlation between the number of documented invasive alien species and land area [37,38]. In Europe, more than 12,000 species of alien plants and animals have been documented [39]. The United States, with a surface area comparable to China, hosts 3,885 invasive species out of 12,981 introduced species records [40]. Moreover, China and US have similar ecogeographic regions; both are major importers and exporters of goods. Notably, a widely-used comprehensive national inventory for invasive species was provided by Xu et al. [13], and the reported number (488) may be a significant underestimate despite the time lag between the introduction and subsequent establishment of non-native species. A verified national checklist was presented on the The Global Register of Introduced and Invasive Species (GRIIS), including 917 records of introduced (alien) and invasive species known to occur in China. [41]. Despite significant efforts and the use of effective prioritization tools to identify potential IAS targets and update quarantine pest lists, the lack of integration for established IAS/quarantine pest lists remains a critical issue (Table 1).

Globally, at least 13,939 plant taxa have become naturalized in regions outside their native distributions [42]. There are 950 plant species that have been naturalized in China, and approximately 400 species are considered invasive [43]. Intensive studies have focused on checklists of alien plants in China [36,44,45,46,47]. However, inventories of other taxonomic groups are not well documented. Additionally, the alien status and introduction dates of many species have rarely been stated and clarified; only a limited number of studies [9,13,34] have annotated the year when an invasive alien species was first detected. Thus, one crucial requirement is the establishment of an information integration system that includes national IAS inventories, promoting the information consolidation of invasive species.

### 2.3. Impact

The impact data of IAS can provide intuitive and specific conclusions and are crucial for supporting invasion-related policies, and critical information regarding the risks or impacts of alien species can inform invasion management and control actions. For example, identifying extremely harmful alien species will guide agencies in taking actions to prevent introductions and contain their spread [48]. However, the gravity of biological invasions has not been generally acknowledged by the public and is undervalued by decision-makers, which is a consequence of the limited knowledge and societal awareness of IAS impacts [49]

Several studies assessing the impact of alien species have emerged in recent years. A recent study showed that the biological invasion of insects costs a minimum of US$76.0 billion per year [2]. As such, the total reported costs of global bio-invasion reached US$1.288 trillion over the past few decades (1970–2017), and the annual cost has steadily increased over time, with no signs of slowing down [50]. Importantly, some effects may be underestimated, as many assessments do not obtain full coverage of the impacted sectors [1], along with biased estimated values due to geographically uneven data. Accordingly, IAS impacts in high-income areas are easily available and simultaneously support decisions regarding the control of invasive species [50,51]. A previous study predicted that China would suffer the highest economic loss in agriculture due to alien invasive pests [52]. The number of alien species for which effects have been reported represents only 3.3% of all introduced species in Asia. Similarly, in China, the number of introduced species is five times higher than those with cost estimates [11], highlighting the absence of impact assessments for a substantial portion of alien species. In particular, given the high GDP, significant trade volume, and the vast numbers of IASs with unreported costs in China, comprehensive and collective assessments of their monetary costs across socioeconomic sectors, taxonomic groups, and geographic regions are still lacking (Table 1).

We extracted records of economic cost estimations from China between 1995 and 2017 based on an established dataset [11], which compiled the actual economic costs of invasions from the InvaCost database [53], incorporating both Chinese and English data. Consistent with our expectations, invasive species, such as *Eichhornia crassipes* (water hyacinth), *Hyphantria cunea* (fall webworms), and *Bursaphelenchus xylophilus* (pine wood nematodes), accounted for a large proportion of the reported analyses. Some analyses have examined how invasions affect ecosystem services and genetic resources, particularly from agricultural, forestry, and fishery perspectives [54]. However, diversifying impacts such as social welfare, urban vegetation, and infrastructure are rarely addressed or quantified. Relevant studies on invasion impacts in China remain insufficient, and existing analyses cover only a small fraction of invasive species, which might lead to taxonomic biases. Since the widely cited analyses [9,55] have been outdated and less representative owing to rapidly escalating biological invasions over decades, quantifying invasion impacts is required to fill the gaps.

## 3. IAS Data Evaluation

**Figure 2:**
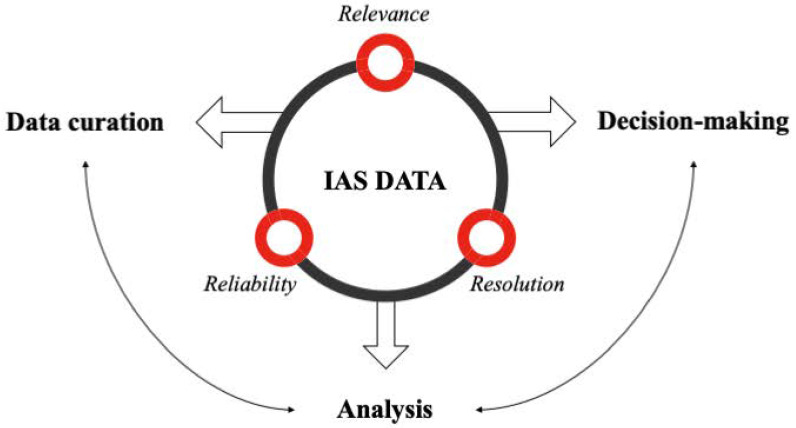
Framework for data quality evaluation of invasive alien species. The processes of data curation (organizing and managing a collection of relevant datasets) and analyses may be essential for evidence-based decision-making. Before that, data should be accessible and analytical. The framework emphasizes three key indicators of IAS data gap analysis that focus on relevance, resolution, and reliability.

Considering the quality of IAS data is crucial during the collection and storage process, we have identified three key indicators: relevance, resolution, and reliability (Figure 2). These indicators can be assessed across multiple dimensions, such as taxonomic, spatial, and temporal aspects. “Relevance” refers to the extent to which the data aligns with the specific needs and objectives. “Resolution” refers to the level of detail and precision within the data, which may range from the resolution of spatial data, the frequency and timeliness of temporal data, to the specification of taxonomic data. “Reliability” refers to the trustworthiness and consistency of the data, ensuring that it is from credible sources and can be confidently used in research, management, and policy-making. Furthermore, the report titled "The Thematic Assessment Report on Invasive Alien Species and Their Control’ from the Intergovernmental Science-Policy Platform on Biodiversity and Ecosystem Services (IPBES)” provides a good example of evaluating gaps in invasive species data and knowledge [56].

## 4. Future Development and Recommendations for China’s IAS Data

### 4.1. Biosecurity Law and Nationwide General Survey of IAS

The Biosecurity Law of the People’s Republic of China has been in effect since 2021, aiming to enhance the management level of biosecurity and establish a national biosecurity coordination mechanism. This law emphasizes national multiagency coordination to enhance the identification, survey, and management of IAS. Additionally, a nationwide general survey of IAS has been conducted in China, providing up-to-date data on biological invasions. On this basis, subsequent research should prioritize data integration and assess the status of alien species, classifying them as invasive where appropriate. In addition to IAS prioritization, efforts should focus on population management and increasing the availability of data in the poluplation dynamics of invasive alien species to ensure effective management strategies. These efforts are expected to bridge significant gaps in IAS distribution, inventories, and impact, delivering reliable information to policymakers and researchers, thereby enabling actions to mitigate the detrimental impacts of IAS.

### 4.2. Balance of IAS Data Security and Sharing

Accurate, detailed, and timely information on biological invasions is necessary; however, certain restrictions on the sharing of phytosanitary data currently hinder the development of cyberinfrastructure [57]. Given the significance and sensitivity of biosecurity data to national security and economic benefits, the misuse or unauthorized disclosure of such data can have serious implications for stakeholders, national security, and international trade. Promoting biosecurity data sharing requires the involvement of various entities and strikes a balance between data security and sharing, while the FAIR principles (i.e., findable, accessible, interoperable, and reusable) provide general guidance for promoting the reuse and integration of scientific data [58,59]. The CARE principles (i.e., collective benefit, authority to control, responsibility, and ethics) are centered around people and purpose, recognizing the pivotal role of data in advancing innovation, governance, and self-determination [60]. Thus, IAS data sharing following FAIR and CARE principles must not harm national biosecurity and abide by the law. 

### 4.3. National Integrated System for IAS

Harmonizing and consolidating reliable data is crucial for converting scattered information into valuable knowledge. China has made significant investments in data collection for IAS; nevertheless, there is still a lack of integrated network information platforms. It is imperative to urgently aggregate and integrate existing data on IAS in China through collaborative efforts. In addition, ensuring quality control is essential to providing accessible data that is reliable and credible, and establishing links to the corresponding information sources for easy tracking is recommended. 

## 5. Conclusions

The emerging trends of IAS have been increasing at unprecedented rates. Deficiency of IAS data may lead to costly and ineffective investment in invasion management. Bridging gaps in regard to data availability and quality to aid assessment, survey and control actions of IAS is an urgent but challenging task in the long term. This study proposes a conceptual framework for the required IAS data in order to trigger more concern about data collection, storage, and curation. Main biological invasion data gaps of distribution, inventories, and impact in China is illustrated. Collaboration between the scientific and management sectors is essential for delivering more fruitful outcomes and ensuring that the management resources are allocated towards effective objectives.

## Figures and Tables

**Table 1:** Key data gaps regarding the distribution, inventories, and impacts of invasive alien species in China are identified and collated. These gaps are expected to be addressed through nationally coordinated research and development as public awareness increases over time.

Data Type	Current Issues	Gaps	Ongoing Efforts
Distribution	Lack of georeferenced data at detailed temporal/spatial scales.	- Inaccuracy - Inadequate data	Systematic surveys and ongoing monitoring are currently underway.
Inventories	Insufficient documentation of introduced species.	- Outdated - Incomplete or underestimated	Joint efforts are underway to update the inventory.
Impact	Evidence of impacts is fragmentary.	- Sporadic research across species - Limited estimations on impact sectors - Underestimated	We are working on the consolidation of impact data.

## Data Availability

No new data were created or analyzed in this study.
